# Deciphering human decision rules in motion discrimination

**DOI:** 10.3758/s13414-021-02327-9

**Published:** 2021-07-08

**Authors:** Jinfeng Huang, Alexander Yu, Yifeng Zhou, Zili Liu

**Affiliations:** 1 Department of Psychology, Hebei Normal University, Shijiazhuang, China; 2 Hefei National Laboratory for Physical Sciences at Microscale, School of Life Science, University of Science and Technology of China, 230027 Hefei, Anhui China; 3 State Key Laboratory of Brain and Cognitive Science, Institute of Biophysics, Chinese Academy of Sciences, 100101 Beijing, China; 4 Department of Computer Science, University of California, Los Angeles, USA; 5 Department of Psychology, University of California, Los Angeles, USA

**Keywords:** Same-different, Motion discrimination, ROC, Signal detection theory

## Abstract

We investigated the eight decision rules for a same-different task, as summarized in Petrov (*Psychonomic Bulletin & Review, 16*(6), 1011–1025, 2009). These rules, including the differencing (DF) rule and the optimal independence rule, are all based on the standard model in signal detection theory. Each rule receives two stimulus values as inputs and uses one or two decision criteria. We proved that the false alarm rate *p*(*F*) ≤ 1/2 for four of the rules. We also conducted a same-different rating experiment on motion discrimination (*n* = 54), with 4^∘^ or 8^∘^ directional difference. We found that the human receiver operating characteristic (ROC) spanned its full range [0,1] in *p*(*F*), thus rejecting these four rules. The slope of the human *Z*-ROC was also < 1, further confirming that the independence rule was not used. We subsequently fitted in the four-dimensional (*p*_*AA*_, *p*_*AB*_, *p*_*BA*_, *p*_*BB*_) space the human data to the remaining four rules—DF and likelihood ratio rules, each with one or two criteria, where *p*_*XY*_ = *p*(responding “different” given stimulus sequence *XY*). We found that, using residual distribution analysis, only the two criteria DF rule (DF2) could account for the human data.

## Introduction

In psychophysics, the same-different task is a basic experimental design that allows investigation of a participant’s sensitivity *d*^′^ and bias in the context of signal detection theory (SDT). This task has the virtue of being straightforward for a participant to understand. For example, in motion direction discrimination, a participant can easily understand what it means for two directions to be the same or different. In comparison, when such discrimination is designed in a two-alternative forced-choice (2AFC) task, it is less intuitive for the participant to understand whether the directional change is clockwise or counter-clockwise, particularly if the average direction varies from trial to trial (Liang, Zhou, & Liu, 2016).

The flip side of the same-different design, however, is that it is difficult to recover *d*^′^. Specifically, *d*^′^ recovery from “same” or “different” responses depends on the decision rule a participant has presumably used. In the literature, there are two classic decision rules (or models) (MacMillan & Creelman, 2005), with all existing rules summarized in Petrov (2009). The first is called the independence rule, which gives rise to optimal performance. Using this rule, the observer determines without bias whether the first stimulus belongs to category *A* or *B*; then, independently, determines whether the second stimulus belongs to *A* or *B*; and finally, the same or different decision is made accordingly. There are three mathematical consequences of this optimal rule, the first two of which are shown in MacMillan and Creelman (2005), and the third proved in the current study. The first consequence is that the proportion of responding “different” when the stimulus sequence is *AB*, denoted here as *p*_*AB*_, is equal to *p*_*BA*_. The next consequence, under the assumption of the standard SDT model of two normal distributions *d*^′^ apart, is that the receiver operating characteristic in *Z*-coordinates (*Z*-ROC) is linear with a slope = 1. The third is that the false-alarm rate *p*(*F*), defined as the probability of deciding “different” given *AA* or *BB* stimuli, or *p*(*F*) = *p*(“different”|*AA* ∪ *BB*), cannot be greater than 1/2. We prove *p*(*F*) ≤ 1/2 in the Appendix and demonstrate its usefulness in model selection.

The second classic decision rule is called the differencing (or DF) rule, which is suboptimal. In this model, the observer takes the difference between the two stimuli in a trial. If the magnitude is greater than some pre-set threshold, the response will be “different”. Otherwise, it will be “same”. This rule, with a single threshold, also leads to *p*_*AB*_ = *p*_*BA*_, *p*_*AA*_ = *p*_*BB*_ and a linear *Z*-ROC, but the *Z*-ROC slope is < 1.

If either of the two rules above is used in a same-different task, the underlying *d*^′^ can be recovered from the experimental data, under the assumption of the standard SDT model. In fact, the same *d*^′^ should be recoverable regardless of the specific experimental task used, be it same-different, yes-no, or 2AFC. There are studies in the literature that confirmed such independence on experimental designs from *d*^′^ recovery. These studies include taste discrimination (Hautus & Irwin, 1995), synthetic vowel discrimination (MacMillan, Goldberg, & Braida, 1988), line-length discrimination (Chen & MacMillan, 1990), and auditory frequency discrimination (Creelman & MacMillan, 1979).

In addition to the independence and differencing rules that an observer may use, there were other studies that aimed to characterize what rules human participants may use in a variety of same-different tasks (DeCarlo, 2013; Irwin & Hautus, 1997). Insightfully, Petrov (2009) pointed out that the four experimental measures, *p*_*XY*_, where *X*, *Y* ∈{*A*, *B*}, contained rich information about what decision rules participants may have used, and should not be lumped together immediately into *p*(*F*) and *p*(*H*) (the hit rate) without examining the equality relationship between *p*_*AB*_ and *p*_*BA*_, and *p*_*AA*_ and *p*_*BB*_. Petrov (2009), using motion discrimination as an example, summarized a set of eight symmetry-based decision rules that took into consideration the two equality relationships above. Symmetry here refers to unchanged decision making when *A* and *B* are exchanged. Table 1 provides a summary of these eight rules, organized into four decision models with either one or two parameters, along with the special cases.

**Table 1:** The four decision models with two parameters each, as summarized in Petrov (2009); the model policies; and their special cases

Model	Model policies	Special cases
Covert classification	The two stimuli are classified independently: “A” if *x* < −*k*_1_, “B” if *x* > *k*_2_, and “ambiguous” otherwise; where − *k*_1_ ≤ *k*_2_. The final response will be “different” *iff* the two stimuli are unambiguous and different. Otherwise, the response will be “same”.	When *k*_1_ = *k*_2_, it is called CC with two symmetric criteria (CC2s). When − *k*_1_ = *k*_2_ = *k*, it is called CC with one criterion (CC1). When this *k* = 0, it is the optimal independence model.
Differencing	“Same” if − *k*_1_ ≤ *x*_2_ − *x*_1_ ≤ *k*_2_, “different” otherwise.	When *k*_1_ = *k*_2_, it is differencing model with 1 criterion.
Likelihood ratio	“Same” if *L*_*d*/*s*_ < *β*_1_ where *d* means *x*_1_ = *A*, *x*_2_ = *B* or if *L*_*d*/*s*_ < *β*_2_ where *d* means $\left (x_{1} = B, x_{2} = A\right .$; “different” otherwise. *β*_1_, *β*_2_ > 0.	When *β*_1_ = *β*_2_, it is likelihood ratio model with 1 criterion *β*. When *β* = 1, it is the optimal model.
Reverse classification	“Different” when *x*_1_ < −*k*_1_ (labeled as “A”) and *x*_2_ > *k*_1_ (labeled as “B”), or when *x*_1_ > *k*_2_ (labeled as “B”) and *x*_2_ < −*k*_2_ (labeled as “A”); where *k*_1_, *k*_2_ ≥ 0. “Same” otherwise.	When *k*_1_ = *k*_2_, it is called reverse classification with two symmetric criteria (RC2s).

Even with these more general rules included as candidates, however, Petrov (2009) showed that the decision rules used by his participants in motion discrimination were not completely determined. This ambiguity is due in part to the binary “same” or “different” responses used in the task, with each participant contributing only a single datum point in the ROC space. As will be proved in this study, for four of the eight total decision rules and regardless of where the decision criteria are placed, the false-alarm rate *p*(*F*) ≤ 1/2 provides a simple and powerful mathematical constraint for model testing. A rating, rather than a binary, same-different experiment offers an opportunity to span the *p*(*F*) (along with *p*(*H*)) in the full range of [0,1], thereby providing the possibility of testing whether or not human *p*(*F*) > 1/2.

The present study used such a same-different rating experiment to generate empirical four-dimensional “ROC”s, namely ROC equivalent in the *p*_*XY*_(*X*, *Y* ∈{*A*, *B*}) space rather than the two-dimensional (*p*(*F*),*p*(*H*)) space, to test all eight candidate models of the same-different task. Our results indicated that the four models with *p*(*F*) ≤ 1/2 could not explain human data. It should be noted that the *p*(*F*) ≤ 1/2 does not depend on the prior probabilities of *AA* and *BB* trials that *p*(*F*) is derived from, although in our human experiment we set the prior *p*(*XY* ) = 1/4. Even within the range of *p*(*F*) ≤ 1/2, some of the mathematical conjectures in Petrov (2009) were empirically disconfirmed, attesting to the value of model fitting.

The four remaining models are the likelihood ratio and differencing models with either one or two decision criteria. Regarding the likelihood ratio rule, on one hand, Petrov (2009) was “doubtful that human observers have the requisite knowledge and processing power to implement” it (p.1012). On the other hand, the various Bayesian observer models (Knill, 1996; Maloney and Mamassian, 2009) largely boil down to a likelihood ratio model in the simple case of our current study. In van den Berg, Vogel, Josic, & Ma (2012) and Shen and Ma (2016), when these likelihood ratio type optimal models were pit against suboptimal models such as differencing models, the human data were better explained by the optimal models. However, these studies did not use motion discrimination, and our current study would test the generality of this optimality hypothesis. To anticipate, our study indicated that the data could be better accounted for by the suboptimal differencing model with two parameters than the optimal likelihood ratio models.

## Same-different rating experiments

### Stimuli and task

**Fig. 1:**
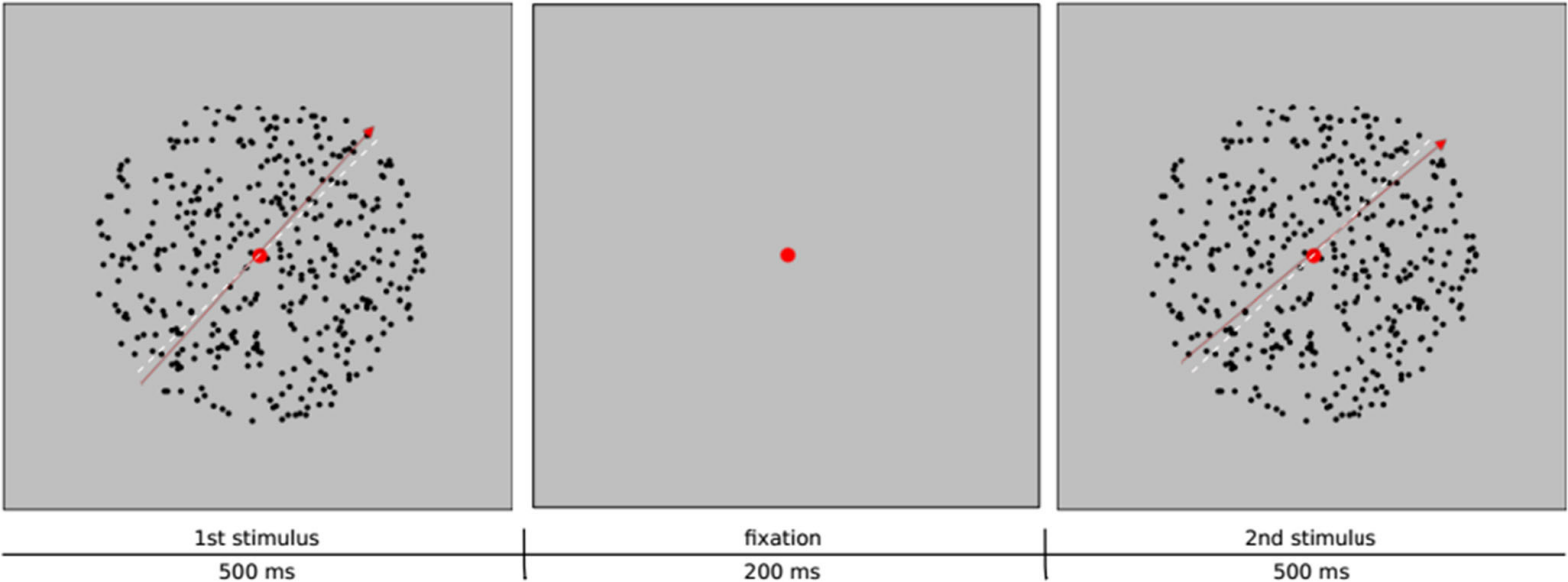
Illustration of one trial in the same-different task. During each trial, the participant saw two stimuli sequentially, each lasted for 500 ms, with an inter-stimulus interval of 200 ms. The participant fixated at the central red disk and decided whether the two motion directions were the same or different by choosing from a six-point rating scale, namely, “surely same,” “same,” “maybe same,” “maybe diff,” “diff,” and “surely diff” (diff = different)

The experiment was a two-interval same-different rating task. In each trial, two random-dot motion stimuli were presented sequentially (Fig. 1), and participants decided whether the two motion directions were the same or different, on a six-point rating scale. Specifically, within a circular aperture of 8^∘^ in diameter (262 pixels) and in gray background (22.0 *cd*/*m*^2^), 400 black random dots (0.0 *cd*/*m*^2^) moved along a single direction with a speed of 10^∘^/*s*. Each dot was 0.09^∘^ in size (a square of 3 × 3 pixels). A central red disk served as the fixation, which was 0.5^∘^ in visual angle in diameter (16 pixels), with a luminance of 5.6 *cd*/*m*^2^.

Each stimulus lasted for 500 ms, and the inter-stimulus interval was 200 ms. The prior probability of *p*(*XY* ) = 1/4. After the second motion stimulus, a six-point rating scale was shown with the following texts at the scale: “surely same,” “same,” “maybe same,” “maybe diff,” “diff,” and “surely diff” (diff = different). Participants used a computer mouse to click on the corresponding label to respond. Trial-wise feedback was provided by a computer beep to a correct response.

In a blocked design, two reference motion directions, ± 45^∘^ (0^∘^ was upward), were selected. The directional difference in each trial, when different, was either ± 4^∘^ or ± 8^∘^. As an example, when the reference direction was 45^∘^ and the difference was ± 8^∘^, the two directions were randomly and independently sampled from the following two directions: 41^∘^ and 49^∘^. Each participant was assigned with only one reference direction (− 45^∘^ or 45^∘^) and one directional difference (4^∘^ or 8^∘^). There were ten blocks of 72 trials. The experiment took close to an hour per participant.

### Participants

Fifty-four students (16 females) from the University of Science and Technology of China (USTC), City of Hefei, participated. They were 21 to 31 years of age (23.6 ± 0.3). Our research protocol was approved by the Ethics Committee of USTC and in accordance with the guidelines of the Declaration of Helsinki. Written informed consent was also obtained from each participant. Participants were unaware of the purposes of the study, and had normal or corrected-to-normal visual acuity via acuity measurement prior to the experiment.

Of the 54 participants, 28 were in the 4^∘^ discrimination task, and 26 the 8^∘^ task.

### Pre-training

The 4^∘^ discrimination task was studied first. The 28 participants were randomly assigned to one of the two reference directions: ± 45^∘^. Before the actual experiment started, they first practiced the task with 4^∘^ directional difference along their assigned directions. Author JH as the experimenter ensured that every participant clearly understood the task. Each participant practiced 61 trials on average.

After the 4^∘^ experiment, it was found that some participants’ accuracies were close to chance. Consequently, 26 additional participants were recruited to the 8^∘^ task. Each participant first practiced 12^∘^ directional discrimination along their assigned direction (− 45^∘^ or 45^∘^), with 14 trials on average. They then practiced the 8^∘^ directional discrimination along their assigned direction, with 129 trials per participant on average. Author JH again worked with every participant to ensure that they clearly understood the task.

### Apparatus

The stimuli were displayed on a 17-inch Sony Multiscan G220 monitor, with a resolution of 1024 × 768 pixels, and a 100-Hz refresh rate. The experiment used MatLab software (MathWorks Corp., Natick, MA, USA) with Psychophysics ToolBox 3 (Brainard, 1997; Pelli, 1997). Participants sat in a dim room and viewed the stimuli binocularly from 60 *cm* away. A chin rest was used to stabilize the participant’s head during the experiment.

### Human behavioral results

To provide an intuitive measure of the participants’ performance, we first computed the accuracy for each participant by categorizing the six-point rating responses into binary responses using the middle criterion. The mean accuracies for the 4^∘^ and 8^∘^ discrimination were 0.55 ± 0.01 (standard error) and 0.74 ± 0.01, respectively.

**Fig. 2:**
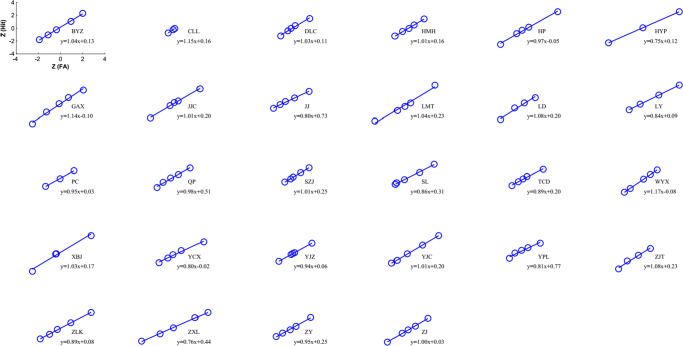
Individual participants’ ROCs in *Z*-coordinates and their linear fittings for the 4^∘^ discrimination. In each panel, the fitted linear equation is shown

**Fig. 3:**
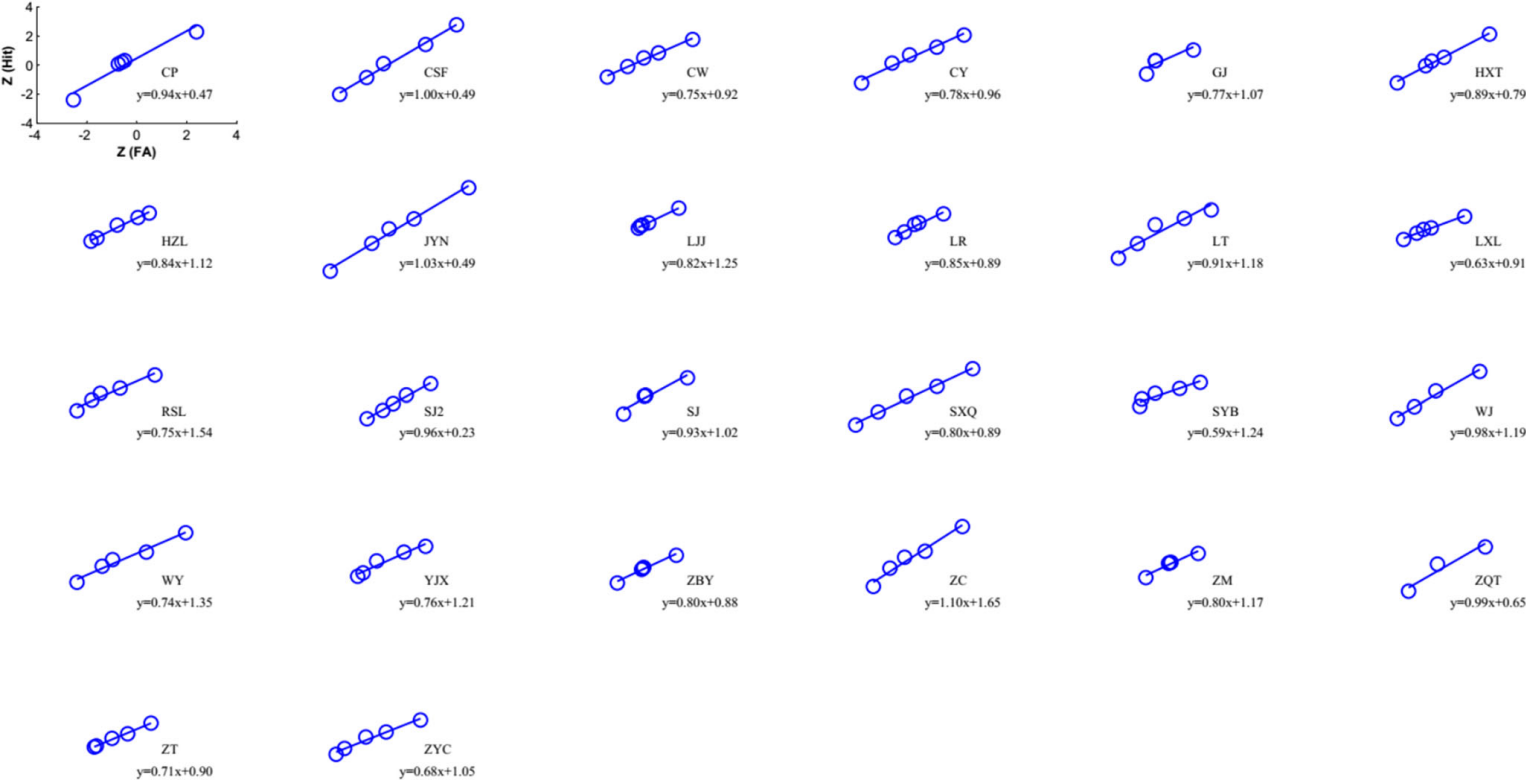
Similar to Fig. 2, individual participants’ ROCs in *Z*-coordinates and their linear fittings for the 8^∘^ discrimination are shown

We then used the six-point rating data to obtain five pairs of (*p*(*H*),*p*(*F*)) and plotted the *Z*-ROC for each participant. Following the conventional correction method to avoid infinity (Wickens, 2002), we added 1/2*n* if a participant’s mean rating was 0, and − 1/2*n* if it was 1, where *n* = 720 was the number of trials per participant. Figures 2 and 3 show the individual participants’ ROCs and their linear fittings using the total least square (TLS) method (Golub & Van Loan, 1980; Wickens, 2002; Liu, Yang, & Intraub, 2016). The mean slope for the 4^∘^ discrimination was 0.96 ± 0.02, which was not significantly different from 1 (*t*(27) = − 1.66,*p* = 0.11, two-tailed). Upon a closer look, however, we found that some of the participants were at chance, whose ROC would be *Z*(*H*) = *Z*(*F*) with a slope = 1. The lowest accuracy was 0.48. We accordingly assumed that 0.52 should also be considered as random variation from the chance of 0.50, and excluded the participants whose accuracies were ≤ 0.52. As a result, ten of the 28 participants were excluded.^1^ The mean slope of the remaining 18 participants was 0.92, and was significantly smaller than one (*t*(17) = 3.00,*p* = 0.008).

In the 8^∘^ condition (where all participants were above chance), the mean slope was 0.84 ± 0.02, and was significantly smaller than one (*t*(25) = 6.49,*p* = 8.58 × 10^− 7^). Taken together, the slope results from the 4^∘^ and 8^∘^ conditions suggest that the discrimination could not possibly have used the covert classification rule with one parameter (CC1), of which the optimal independence rule is a special case when the criterion is unbiased.

It should be noted that the hypothesis testing of slope = 1 as predicted by the CC1 model in this section is different from model testing in the next section. Here, the human data are *Z*(*H*) and *Z*(*F*), and the slope = 1 hypothesis is a mathematical result from the independence rule. In the next section, the human data will be *p*_*XY*_ and the model data will also be *p*_*XY*_ derived from the specific model assumptions. In this sense, the hypothesis testing in the current section and the next will be largely independent of each other. Therefore, if the CC1 model is again tested in the next section with model fitting and shown not to account for human data, then we will have converging evidence that the *Z*-ROC slope method and model fitting method gave rise to consistent results. Hence we will start the next section by verifying the results obtained in the current section, but only using the human *p*_*XY*_ data when $p(F) = \frac {1}{2} \left (p_{AA} + p_{BB} \right ) \leq 1/2$ to ensure fairness.

## Fitting the human data with the one- and two-parameter differencing and likelihood ratio rules

We proved in the Appendix that the false-alarm rate *p*(*F*) ≤ 1/2 for the covert classification (CC) and reverse-classification (RC) models, regardless if the models have one or two parameters, and regardless of how the parameters are chosen. Since the human *p*(*F*) could exceed 1/2 (or *Z*(*F*) > 0, Figs. 2 and 3), the four models above could not account for the human data.

For the remaining four models, namely differencing and likelihood ratio models with one or two parameters, there are the following two ways to fit them with human data. The first is suggested by Petrov (2009), which is to use *χ*^2^ null hypothesis testing to verify the following equalities: *p*_*AA*_=*p*_*BB*_ and *p*_*AB*_ = *p*_*BA*_, and categorize accordingly which participants’ data qualitatively fit which models. However, this approach of categorization using null hypothesis testing has to rely on a fixed *α* value (e.g., *α* = 0.05) that is somewhat arbitrary (Rozeboom, 1960). For example, *p*_*AA*_ = *p*_*BB*_ is deemed acceptable in a *χ*^2^ analysis if *p* = 0.052, but is rejected if *p* = 0.048, although the two cases can be practically the same.

Alternatively, one can fit all the *p*_*AA*_, *p*_*BB*_, *p*_*AB*_, and *p*_*BA*_ human data, rather than human data only consistent with the *χ*^2^ test per Petrov (2009), to a specific model. Here, the fitting will be similar to the *Z*-ROC fitting in the last section using the TLS method (Golub & Van Loan 1980; Wickens, 2002; Liu et al. 2002) , except that it will be in the 4-D *p*_*XY*_ space.^2^

In what follows, we will fit all human data with the likelihood ratio and differencing models with one and two parameters. We will check whether the residuals are evenly distributed across different confidence levels and across the four dimensions of *p*_*XY*_ (Kellen and Singmann, 2016). Afterwards, to seek converging evidence, we will also check $p_{AA} \left (\begin {array}{ll}{=}\\{\neq }\end {array}\right ) p_{BB}$ and $p_{AB} \left (\begin {array}{ll}{=}\\{\neq }\end {array}\right ) p_{BA}$ for model selection according to Petrov (2009).

But first, we will verify the effectiveness of this approach of ROC model fitting by checking whether such model fitting gives rise to results consistent with the results independently obtained in “Human behavioral results”, namely the human *Z*-ROCs had a mean slope smaller than one.

### Verifying the effectiveness of the model fitting approach

In “Human behavioral results”, we compared the slopes of human Z-ROCs with that predicted by the covert classification model with one parameter (CC1), of which the independence rule is a special case. In that comparison, we found that the slopes of human *Z*-ROCs were different from the model prediction, thereby rejected the CC1 model as a candidate.

We now independently verify if CC1 model fitting in the 4-D *p*_*XY*_(*X*, *Y* ∈{*A*, *B*}) space would yield similar results. Since the model’s *p*(*F*) ≤ 1/2, we used human data whose *p*(*F*) ≤ 1/2 also. We further verified the CC model with two parameters (CC2a), of which CC1 is a special case. For both models, and for all the 26 8^∘^ participants and the 18 4^∘^ participants who were above chance, the fittings were rejected because the residuals of the model fitting were not evenly distributed across the four dimensions of *p*_*XY*_. This indicates that the 4-D model fitting was consistent with the independent ROC slope analysis. The details of this verification are in Appendix C.

In what follows, we will apply the similar model fitting to the remaining four models, in two aspects. (1) We will measure the residuals across the *p*_*XY*_’s and rating scale. (2) We will check the *relative* residual distributions across these two dimensions, because an uneven distribution would indicate poor fitting.

Regarding (1), we first calculated *χ*^2^ between model prediction and each participant’s data, and then calculated the cumulative *χ*^2^ across all participants, for the 4^∘^ and 8^∘^ conditions, respectively. Given the large degrees of freedom (since each participant across ten sessions contributed 240 numbers), the resultant *χ*^2^ distribution could be well approximated by a normal distribution. The discrepancy between the human data as a whole and each model was highly significant (*Z* ≥ 9.25).

That is to say, neither of these four models was a good fit to the human data in terms of absolute residue magnitudes. This is perhaps not very surprising for the following two reasons.

1. Petrov (2009) qualitatively determined which participant’s data were consistent with which model, checking whether or not *p*_*AA*_=*p*_*BB*_ and *p*_*AB*_=*p*_*BA*_ (see below). In our case, only a certain proportion of participants’ data were consistent with each of the four models. As a result, when all participants’ data were considered, the majority of the participants’ data violated one or both of equations above, which ensured a large discrepancy in *χ*^2^.
2. All participants were inexperienced (albeit with pre-experiment practice). This in itself made it possible for the data to have large residuals.

In the remainder of this section, we will focus on (2), whether or not residuals were evenly distributed across the *p*_*XY*_’s and rating scale.

### Fitting human data to the likelihood ratio model with one parameter (LR1)

By definition, *p*_*XY*_ = *p*(responding “different”|*XY* ), where *X*, *Y* ∈{*A*, *B*}. From Irwin and Hautus (1997),^3^ given that $p(H) = \frac {1}{2} \left (p_{AB} + p_{BA} \right )$ and $p(F) = \frac {1}{2} \left (p_{AA} + p_{BB} \right )$ (under the assumption that the prior *p*(*XY* ) = 1/4); and that *p*_*AA*_ = *p*_*BB*_, *p*_*AB*_ = *p*_*BA*_ for the LR1 model, we have:

1 $$ \begin{array}{@{}rcl@{}} \text {When } \beta & >& 1, \\ p_{AA} = p_{BB} = p(F) &=& 1- {{\Sigma}}_{i=1}^{2} \left( 1 - {\Phi} \left( \frac{ln \left( \beta \right)}{d'} + (-1)^{i} \frac{d'}{2} \right) \right)^{2},\\ p_{AB} = p_{BA} = p(H) &=& 1 - 2 {\Pi}_{i=1}^{2} \left( 1 - {\Phi} \left( \frac{ln \left( \beta \right)}{d'} + (-1)^{i} \frac{d'}{2} \right) \right).\\ \text {When } \beta & \leq & 1, \\ p_{AA} = p_{BB} = p(F) &= &2 {\Pi}_{i=1}^{2} \left( 1 - {\Phi} \left( \frac{-ln \left( \beta \right)}{d'} + (-1)^{i} \frac{d'}{2}\right) \right), \\ p_{AB} = p_{BA} = p(H) &= &{\Sigma}_{i=1}^{2} \left( 1 - {\Phi} \left( \frac{-ln \left( \beta \right)}{d'} + (-1)^{i} \frac{d'}{2}\right) \right)^{2}, \end{array} $$

where Φ(⋅) is the cumulative distribution function (CDF) of a normalized Gaussian.

**Fig. 4:**
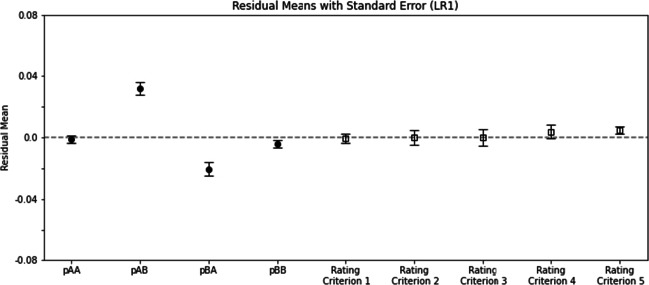
Residual means from using the LR1 (likelihood ratio rule with one parameter) to fit the 54 participants’ data. These means are plotted across the four dimensions of *p*_*XY*_, *X*, *Y* ∈{*A*, *B*} and across the five rating criteria. *Error bars* represent standard error of the mean

Figure 4 shows the means of residuals of fitting the 54 participants’ data using the LR1 model. The residual data were analyzed in a two-way ANOVA with *p*_*XY*_ as one factor and rating criterion as the other factor. The main effect of *p*_*XY*_ was significant, *F*(3,159) = 9.83,*p* = 5.53 × 10^− 6^. The main effect of rating criterion was significant, *F*(4,212) = 10.15,*p* = 1.55 × 10^− 7^. The interaction was also significant, *F*(12,636) = 8.39,*p* = 7.62 × 10^− 15^. This means that the residuals were unevenly distributed across different levels of rating, and unevenly in responses to different stimuli. We conclude that the LR1 was not a good candidate fitting the human data.

### Fitting human data to the likelihood ratio rule with two parameters (LR2)

For model LR2, *p*_*AA*_ = *p*_*BB*_, *p*_*AB*_ ≠ *p*_*BA*_. This means that, in Eq. 1, the threshold *β* used for *p*_*AB*_ is different from that used for *p*_*BA*_.

**Fig. 5:**
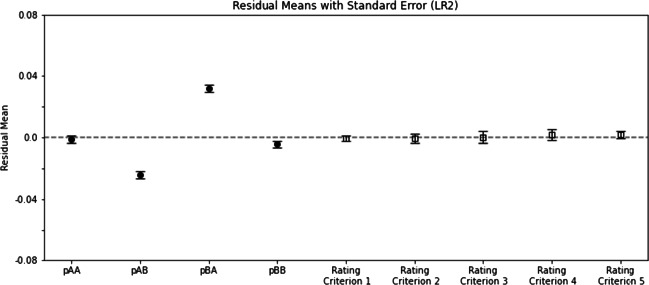
Similar to Fig. 4 except that two-parameter model LR2 was used to fit the human data

Figure 5 shows the fitting results of the LR2 model, similarly plotted as Fig. 4. A similar ANOVA indicated that all effects were significant: the main effect of rating criterion (*F*(4,212) = 2.84,*p* = 0.025); the main effect of *p*_*XY*_ (*F*(3,159) = 27.92,*p* = 1.48 × 10^− 19^); and the interaction (*F*(12,636) = 12.09,*p* = 3.02 × 10^− 24^). These results indicate that the residual distributions were not uniform across different rating criteria and across different *p*_*XY*_ response variables. As can be seen in Fig. 5, the uneven distribution of the residuals across *p*_*XY*_ was particularly pronounced. Consequently, the LR2 model was not a good candidate to explain the human data either.

### Fitting human data to the differencing model with one parameter (DF1)

For model DF1, similar to LR1, *p*_*AA*_ = *p*_*BB*_, *p*_*AB*_ = *p*_*BA*_. We have:

2 $$ \begin{aligned} p_{AA} = p_{BB} = p(F) &= 2 {\Phi} \left( -\frac{k}{\sqrt{2}} \right), \\ p_{AB} = p_{BA} = p(H) &= {\Sigma}_{i=1}^{2} {\Phi} \left( \frac{-k + (-1)^{i} d'}{\sqrt{2}} \right), \end{aligned} $$

where Φ(⋅) is the cumulative distribution function (CDF) of a normalized Gaussian.

**Fig. 6:**
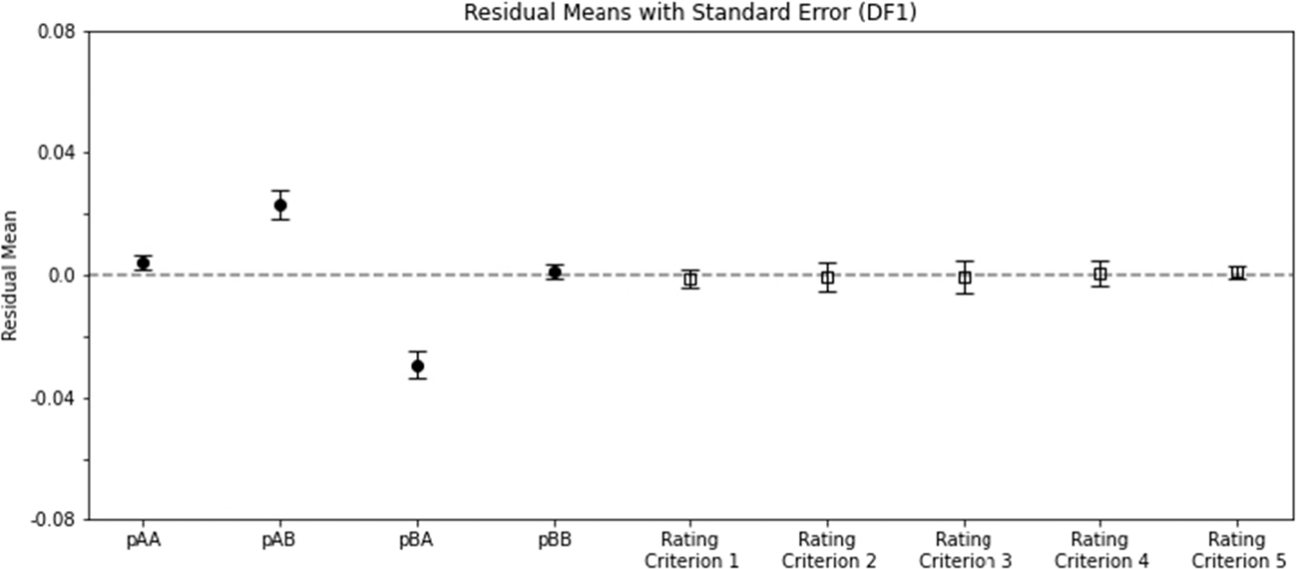
Similar to Fig. 4 except that the fitted model is DF1, the one-parameter differencing rule

Figure 6 similarly shows DF1 model fitting results. A two-way ANOVA on the residuals across rating criteria and across *p*_*XY*_’s yielded the following significant results. The main effect of rating criterion was significant (*F*(4,212) = 2.79,*p* = 0.027). The main effect of *p*_*XY*_ was significant (*F*(3,159) = 8.93,*p* = 1.67 × 10^− 5^). The interaction was highly significant *F*(12,636) = 5.91,*p* = 8.53 × 10^− 10^). These results indicate that the residuals were again unevenly distributed across rating criteria and across *p*_*XY*_’s. Consequently, DF1 was not a good candidate to explain the human data as a whole.

### Fitting human data to the differencing model with two parameters (DF2)

For model DF2, *p*_*AA*_ = *p*_*BB*_ but *p*_*AB*_ ≠ *p*_*BA*_. This means that the threshold *k* used for *p*_*AB*_ in Eq. 2 is different from that used for *p*_*BA*_.

**Fig. 7:**
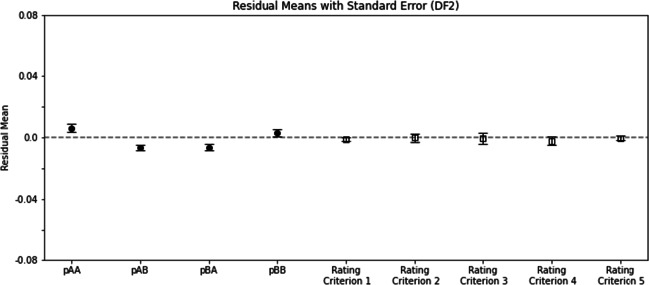
Similar to Fig. 4 except that the fitted model is DF2, the two-parameter differencing rule

A two-way ANOVA on the residuals across rating criteria and across *p*_*XY*_’s yielded the following results, the main effects of which were different from the three models above. Namely, the main effect of rating criterion was not significant (*F*(4,212) = 1.90,*p* = 0.11). Nor was the main effect of *p*_*XY*_ significant (*F*(3,159) = 2.18,*p* = 0.092). The interaction was significant (*F*(12,636) = 5.95,*p* = 6.89 × 10^− 10^). These results indicate that the residuals shared comparable means across different rating criteria, and across different *p*_*XY*_ measures. Note that the comparable means along the dimensions of rating criteria and *p*_*XY*_’s were from all participants’ data, including those whose *p*_*AB*_ ≠ *p*_*BA*_. The significant interaction effect was possibly due to these data with *p*_*AB*_ ≠ *p*_*BA*_. To verify this conjecture, we separately analyzed data that were accepted by the *χ*^2^ test in the residual analysis (n = 23), and found not surprisingly that the two main effects remain nonsignificant (*F*(4,88) = 1.25,*p* = 0.29;*F*(3,66) = 0.99,*p* = 0.40). But the interaction became much weaker also (*F*(12,64) = 1.81,*p* = 0.047) (Fig. 7).

Taken together, and primarily from the lack of main effects in the residual analysis, we conclude that the human data were consistent with the differencing rule with two parameters. Namely, the threshold used in deciding whether the difference was small enough between the two stimuli in a trial depended on the sequence of the two stimuli. In other words, in the decision to respond “same” when −|*k*_1_|≤ *x*_1_ − *x*_2_ ≤|*k*_2_|, there was participant bias that depended on the stimulus sequence.

Assuming that the human participants indeed used the DF2 rule, then the mean of the best fitting *d*^′^s from the 28 4^∘^ participants was *d*^′^ = 1.04 ± 0.09 (standard error). The mean of the best fitting *d*^′^s of the 26 8^∘^ participants was *d*^′^ = 2.40 ± 0.06. Under the standard SDT model, the mean standard deviation of a motion direction in the 4^∘^ condition was 4^∘^/1.04 = 3.85^∘^. In the 8^∘^ condition, this standard deviation was 8^∘^/2.40 = 3.33^∘^. These two estimates were not significantly different (*t*(52) = 1.53,*p* = 0.13). This indicates that the standard deviation of perceiving a motion direction was approximately 3.59^∘^.

## Qualitative model selection per Petrov (2009)

Petrov (2009) proposed to use *χ*^2^ tests to verify $p_{AA} \left (\begin {array}{ll}{=}\\{\neq }\end {array}\right )$ *p*_*BB*_ and $p_{AB} \left (\begin {array}{ll}{=}\\{\neq }\end {array}\right ) p_{BA}$. Qualitative model selection can then be accomplished according to the four possible outcomes (but before considering our new result that *p*(*F*) ≤ 1/2 for some models).

Table 2 shows the number of participants satisfying each of the four equality-inequality cases, along with the candidate decision rules for that case. In the current study, since a rating experiment was used, we applied the *χ*^2^ test per decision criterion. However, since rating data are not completely independent of each other, the degrees of freedom used in the *χ*^2^ test was an overestimate. For this reason, we also binarized the rating data by using the middle criterion, recalculated *χ*^2^ per block per participant, and recategorized the four cases. The results are shown in Table 2 in parentheses.^4^

**Table 2:** The distribution of the number of participants and the candidate decision rules according to the equality relationships of the four stimulus pairs

	Case	No. Participants	Candidate rules
1	*p*_*AA*_ = *p*_*BB*_, *p*_*AB*_ = *p*_*BA*_	11 (17)	DF1, CC2s, LR1
2	*p*_*AA*_ ≠ *p*_*BB*_, *p*_*AB*_ = *p*_*BA*_	4 (4)	CC1, CC2a
3	*p*_*AA*_ = *p*_*BB*_, *p*_*AB*_ ≠ *p*_*BA*_	26 (26)	DF2, RC2a, LR2
4	*p*_*AA*_ ≠ *p*_*BB*_, *p*_*AB*_ ≠ *p*_*BA*_	13 (7)	None

The numbers in parentheses are obtained by collapsing the rating data into “same” “different” binary responses using the middle criterion only. DF1: differencing rule with one criterion; CC2s: covert classification with two symmetric criteria; LR1: likelihood ratio with one criterion; CC1: covert classification with one criterion; CC2a: covert classification with two asymmetric criteria; DF2: differencing rule with two criteria; RC2a: reversed-classification with two asymmetric criteria; LR2: likelihood ratio with two criteria

To summarize from Table 2, out of the 54 participants total and using Petrov’s (2009) qualitative model selection method, data from 74% of the participants could be explained by the differencing (DF) and likelihood ratio (LR) rules (cases 1 and 3), whereas the remaining 26% could not be explained by any rules (cases 2 and 4). If we keep in mind that the null *χ*^2^ hypothesis testing used here with *α* = 0.05 has some degree of arbitrariness, then the qualitative model selection in this section is, broadly speaking, consistent with the quantitative model fitting results in the last section. The overlap between these two sections is that the biased (or two-parameter) differencing rule is a candidate to account for the majority of human data. This conclusion is also consistent with the argument in Petrov (2009) that human participants may not be able to have access to the full details of the optimal likelihood ratio models. The differencing rule as a candidate is also appealing in that executing this rule (taking the difference between two stimuli) is intuitive.

## Discussion

In the current study, we collected data from 54 participants in a same-different rating experiment on motion discrimination, with two levels of directional difference, 4^∘^ and 8^∘^. With all eight models available in the literature, we fitted to each individual participant’s data a four-dimensional “ROC” in the *p*_*XY*_ space, rather than the conventional ROC in the two-dimensional (*p*(*F*),*p*(*H*)) space. We found the following:

- The false alarm rate *p*(*F*) can be proven mathematically to be *p*(*F*) ≤ 1/2 for four of the eight models.
- Since our rating experiment could obtain an ROC with a large range of decision criteria, our human *p*(*F*)’s could exceed 1/2, hence rejecting the four models above, including the covert and reverse classification models (CC and RC), with either one or two parameters. A supplementary, binary same-different experiment, which was otherwise identical to the main experiment, further confirmed that *p*(*F*) could exceed 1/2. This means that participants could indeed position their decision criterion such that *p*(*F*) > 1/2.
- In particular, the well-known optimal model, the independence rule, could be rejected because it is a special case of the covert classification rule with one parameter.
- The differencing rule with two parameters (DF2) well accounted for the human data as a whole, as opposed to the rest of the three models: DF1, LR1, and LR2.

We should qualify that, when we said that the DF2 model accounted for the participants’ data as a whole, we meant that some combinations of the two criteria could well approximate the participants’ data across the full rating criteria. However, we do not understand how in principle a participant’s five rating criteria were chosen in any systematic way, if DF2 was used. In other words, we do not understand how the DF2 model’s two criteria were positioned to give rise to each of the five rating criteria. In fact, we only know that the DF2 model was a candidate to explain the human data. Whether or not the participants actually used this rule or some other yet unknown rule, remains an open question. In this sense, we are still far from understanding the functional mechanism of human decision making in motion discrimination.

Since Petrov (2009) also used motion direction discrimination as an example problem in his binary same-different task, it is informative to compare his results with ours. In his study, among 13 participants total, data from 11 were consistent with DF2, RC2a, and LR2. Since we proved in the current study that *p*(*F*) ≤ 1/2 for RC2a, the two studies converged on DF2 and LR2.^5^

It is interesting to note that, in Petrov (2009), no participant’s data violated both symmetry constraints, such that no participant’s data were in case 4. In our study, if we used the *χ*^2^ null hypothesis test, on average ten out of the 54 participants’ data (or 19%) were in case 4, not explainable by any rules. What might be the discrepancy between the two studies? Petrov (2009) collected the data in a motion discrimination perceptual learning experiment with four training sessions, plus a pre-training test session and a post-training test session. Because his analysis focused on the symmetry constraints of *p*_*AB*_ = *or*≠*p*_*BA*_? and *p*_*AA*_ = *or*≠*p*_*BB*_? for model selection, an increased *d*^′^ as a result of perceptual learning would be unlikely to affect the analysis. His approach has an advantage that participants could have possibly settled into their strategies as a result of the perceptual learning. In comparison, our participants ran only a single session of the experiment. Although these participants went through pre-experiment practice and would not start the experiment until deemed ready, we could not rule out the possibility that some participants were still exploring and switching strategies. That said, however, since our main aim was to fit a 4-D “ROC” with a steady *d*^′^, we could realistically use only a single daily session’s data when *d*^′^ was presumably steady. Compared to perceptual learning studies, our study investigated generic motion direction discrimination with participants who did not go through extensive training, but nevertheless practiced the task prior to the main experiment. In this sense, we believe that our results are informative about how non-expert participants discriminated motion directions.

### Optimal and suboptimal models

Although we have tested all of the eight same-different models in the literature that we are aware of, these models are all based on the standard SDT model and can be certainly extended. To illustrate, van den Berg et al., (2012) studied same-different visual discrimination using an array of oriented ellipses, with the goal of testing whether optimal or suboptimal models better accounted for human performance. Here, since the number of independently varying orientations was greater than two, the standard SDT model no longer applied. Yet, the optimal model could still be constructed that compared the ratio between the posterior probability of all ellipses being identically oriented over that of the ellipse orientations being different. Such a model is mathematically equivalent to the simpler LR1 model in the current study with the criterion *β* = 1. The suboptimal model in van den Berg et al., (2012) calculated pair-wise orientation differences between the ellipses, which is a variation of the DF1 model. These authors found that their optimal model “accurately describes human behavior,” and outperformed those differencing type of models.

Beyond same-different tasks, the optimal Bayesian models (Ma, 2019) can be all boiled down to the likelihood ratio models (LR) in the simple case of the current study. This Bayesian approach offers a broader platform (beyond same-different discrimination) to evaluate perceptual decision making. For example, Shen and Ma (2016) deliberately pit a complex optimal decision rule (which is equivalent to the LR1 model, in the current study’s simpler case) against suboptimal rules (e.g., DF models) such that these models predicted qualitatively different results from the optimal model. It turned out that their optimal model well fit the human data, whereas the simpler but suboptimal models failed to account for the human data. Consequently, their results are supportive of the optimal LR models, although we note that their experiments were neither same-different nor motion discrimination.

In contrast, in the current study on same-different motion discrimination, the suboptimal DF2 model better accounted for the human performance than the optimal LR models. It remains an open question whether this discrepancy is due to different tasks used, or to the complexity of different models. This question regarding optimality is intriguing, and certainly worthy of continued investigation.

## Figures and Tables

**Fig. 8:**
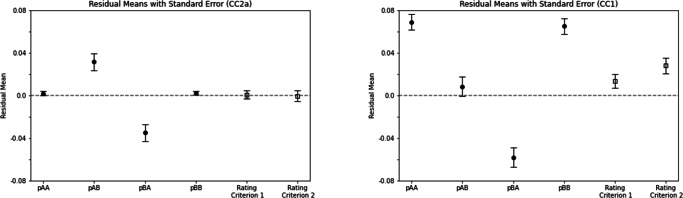
Means of the residuals of model fitting across the four *p*_*XY*_ dimensions (*X*, *Y* ∈{*A*, *B*}) and the two lowest rating criteria (so that *p*(*F*) is ensured to be ≤ 1/2). *Left*: CC2a model fitting residuals. *Right*: CC1 model fitting residuals

**Fig. 9:**
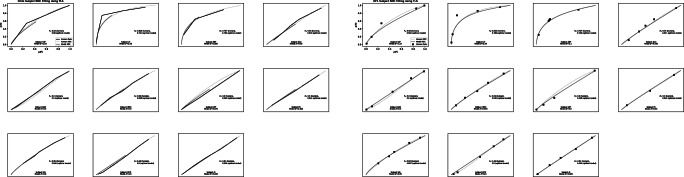
Model comparison between the CC2s (*left*) and DF1 (*right*) models in fitting the human data. The fitted *d*^′^s were consistent with the mathematical prediction in Petrov (2009) that *dCC*2*s*′ < *dDF*1′. The proportion correct, *P*_*c*_, as predicted by this optimal rule, and the actual human *P*_*c*_ are also shown per participant, as in other figures

**Fig. 10:**
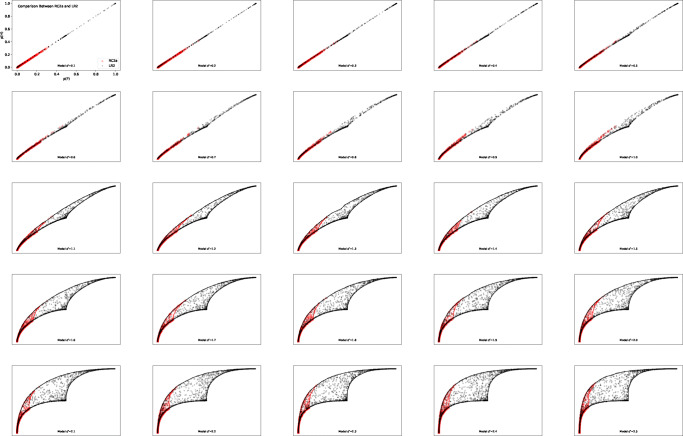
Model comparison between the RC2a (in red) and LR2 models in fitting the human data. Apparently, the RC2a model covers only a limited range of *p*(*F*). Hence, this approximation cannot be close in the full range

**Fig. 11:**
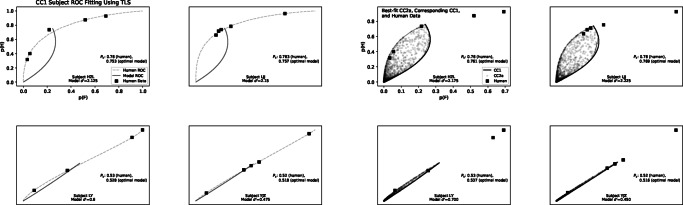
Model fittings for the CC1 (*left*) and CC2a (*right*), with human data as reference. CC1 as a one-parameter model has a single ROC curve. CC2a as a two-parameter model has a cloud of model datum points. CC1 can be considered as a special case of CC2a when *c*_1_ = *c*_2_ and therefore, was predicted to share the same *d*^′^ as CC2a in Petrov (2009). Among the four participants’ data here, the CC1’s *d*^′^ fittings were numerically smaller than the CC2a’s in the top row, and the other way around in the bottom row. (On top of this cloud is the special case of CC2s, when − *c*_1_ = *c*_2_. However, CC2s was not supposed to be able to account for the human data due to the symmetry constraints of human responses)

**Fig. 12:**
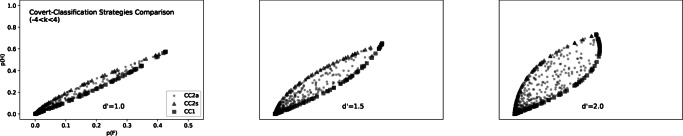
Model fittings with *d*^′^ = 1 (left), *d*^′^ = 1.5 (*middle*), and *d*^′^ = 2 (*right*) between the CC2s (top ROC), CC2a (model data cloud), and CC1 (bottom ROC) in each panel

**Fig. 13:**
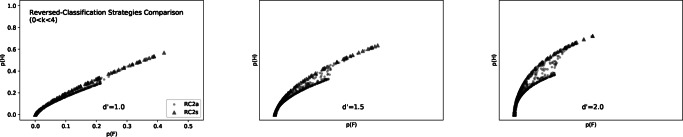
Model fittings with *d*^′^ = 1 (*left*), *d*^′^ = 1.5 (*middle*), and *d*^′^ = 2 (*right*) between the RC2s (top ROC) and RC2a (data clouds) in each panel

## Appendix A: Proof of *p*(*F*) ≤ 1/2 in the RC2a rule

In pages 1014-1015, Petrov (2009) said: “...if the first stimulus is classified as “*B*” when *x*_1_ > *c*_2_, the second stimulus is classified as “*A*” when *x*_2_ < −*c*_2_. If the first stimulus is classified as “*A*” when *x*_1_ < *c*_1_, then second stimulus is classified as “*B*” when *x*_2_ > −*c*_1_. With symmetric criteria (*c*_1_ = −*c*_2_), the resulting rule coincides with the CC2s rule”.^6^

It should be apparent from the above that *c*_1_ < 0 because *c*_1_ ≤−*c*_1_ in order to avoid double labeling (because otherwise, if − *c*_1_ < *c*_1_, then when *x*_1_ < *c*_1_, *x*_2_ can be labeled both as “A” and “B” if − *c*_1_ < *x*_2_ < *c*_1_). It is also apparent that when the first stimulus is classified as *B* correctly or incorrectly, criterion *c*_2_ will be used for both the first and second stimuli. Therefore, when the response is correct, *p*(“first response is B”$|B) =b_{B1} =1 - {\Phi } \left (c_{2} - \frac {d'}{2} \right )$. When the response is incorrect, *p*(“first response is B”$|A) = b_{A1}=1 - {\Phi } \left (c_{2} + \frac {d'}{2} \right )$.

Likewise, if the first stimulus is classified as *A* (centered at -$\frac {d'}{2}$) correctly or incorrectly, criterion *c*_1_ is used for both the first and second stimuli. Therefore, when the response is correct, *p*(“first response is A$|A ) = a_{A1}={\Phi } \left (c_{1} + \frac {d'}{2} \right )$. When the response is incorrect, *p*(“first response is A”$|B) = a_{B1} = {\Phi } \left (c_{1} - \frac {d'}{2} \right )$.

We now consider the false alarm rate *p*(*F*) by definition,^7^

A.1 $$ \begin{array}{@{}rcl@{}} p(F) &= &p(\text {``responding different''}|AA \cup BB)\\ &= &p(\text {``responding different''}|AA)p(AA|AA \cup BB)\\ && + p(\text {``responding different''}|BB)p(BB|AA \cup BB)\\ &= & \frac{1}{2} \left( p \left( \text {``responding different''}|AA \right)\right.\\ &&\left.+ p \left( \text {``responding different''}|BB \right) \right) \\ &=& \frac{1}{2} \left( a_{A1} b_{A2}+b_{A1} a_{A2} + a_{B1} b_{B2} + b_{B1} a_{B2} \right). \end{array} $$

Let us look at the first two terms in the parenthesis, *a*_*A*1_*b*_*A*2_ + *b*_*A*1_*a*_*A*2_. Recall *a*_*A*1_ means that “responding A” when the first stimulus is A, that *c*_1_ is used when the first stimulus is responded as “A,” and that *c*_2_ is used when the first stimulus is responded as “B”.

A.2 $$ \begin{array}{@{}rcl@{}} a_{A1} b_{A2} &=& {\Phi} \left( c_{1}+\frac{d'}{2} \right) \left( 1 - {\Phi} \left( -c_{1} - \left( -\frac{d'}{2} \right) \right) \right),\\ b_{A1} a_{A2} & =& \left( 1 - {\Phi} \left( c_{2} + \frac{d'}{2} \right) \right) {\Phi} \left (-c_{2} - \left( -\frac{d'}{2} \right) \right).\\ \text{Note that } a_{A2} &=& {\Phi} \left (-c_{2} - \left( -\frac{d'}{2} \right) \right) = 1 - {\Phi} \left (c_{2} - \frac{d'}{2} \right), \\ \text{ and } b_{A2} &= &1 - {\Phi} \left( -c_{1} - \left( -\frac{d'}{2} \right) \right) = {\Phi} \left( c_{1} - \frac{d'}{2} \right). \end{array} $$

Similar derivation, or better yet, the symmetry consideration, gives us (as it should, given the necessary condition for RC2a)

A.3 $$ p(\text {responding ``different''}|BB) = p(\text {responding ``different''}|AA). $$

Hence,

A.4 $$ \begin{array}{@{}rcl@{}} p(F)& =& {\Phi} \left( c_{1} + \frac{d'}{2} \right) {\Phi} \left( c_{1} - \frac{d'}{2} \right) \\&&+ \left( 1 - {\Phi} \left( c_{2} + \frac{d'}{2} \right) \right) \left( 1 - {\Phi} \left( c_{2} - \frac{d'}{2} \right) \right). \end{array} $$

Note in the derivations above there might not appear a need for *c*_1_ ≤ *c*_2_. However, since *a*_*A*1_ = *b*_*B*2_ and *b*_*A*1_ = *a*_*B*2_, it is necessary that *c*_1_ ≤ 0 ≤ *c*_2_ to avoid overlap labeling. We next prove that *p*(*F*) ≤ 1/2.

A.5 $$ \begin{aligned} \because &  c_{1} \leq 0,\\ \therefore & {\Phi} \left( c_{1} + \frac{d'}{2} \right) \leq 1 - {\Phi} \left( c_{1} - \frac{d'}{2} \right). \end{aligned} $$

This means that, starting at *c*_1_ ≤ 0, the right tail of the normal distribution $N \left (-\frac {d'}{2}, 1 \right )$ has a greater area than the left tail of the normal distribution $N \left (\frac {d'}{2}, 1 \right )$.

A.6 $$ \begin{aligned} \therefore &  {\Phi} \left( c_{1} + \frac{d'}{2} \right) {\Phi} \left( c_{1} - \frac{d'}{2} \right) \leq \left( 1 - {\Phi} \left( c_{1} - \frac{d'}{2} \right) \right) {\Phi} \left( c_{1} - \frac{d'}{2} \right) \leq \frac{1}{4}. \end{aligned} $$

Using left-right symmetry, it can be similarly proved that

A.7 $$ \left( 1 - {\Phi} \left( c_{2} + \frac{d'}{2} \right) \right) \left( 1 - {\Phi} \left( c_{2} - \frac{d'}{2} \right) \right) \leq \frac{1}{4}. $$

Therefore,

A.8 $$ \begin{array}{@{}rcl@{}} p(F) &= &{\Phi} \left( c_{1} + \frac{d'}{2} \right) {\Phi} \left( c_{1} - \frac{d'}{2} \right) \\&&+ \left( 1 - {\Phi} \left( c_{2} + \frac{d'}{2} \right) \right) \left( 1 - {\Phi} \left( c_{2} - \frac{d'}{2} \right) \right)\\ & \leq &\frac{1}{2}. \end{array} $$

## Appendix B: Proof that *p*(*F*) ≤ 1/2 in CC2a, CC2s, and CC1

To ensure absolute clarify regarding how the CC2a (covert classification with two asymmetric criteria) works according to Petrov (2009), and to ensure that we have not added any extra assumptions or conditions, we first quote the relevant definition from Petrov (2009), page 1024: The CC2a decision rule is a generalization of CC1 that uses three covert categories: “A,” “B,” and “ambiguous”. This requires two criteria *c*1 ≤ *c*2... The observer responds “different” iff one stimulus is unambiguously classified “A” and the other “B”.

It should be emphasized that the condition *c*1≤*c*2 for the two criteria was specified in Petrov (2009), and not enforced by us. It should also be emphasized that the “different” response is definitive and unambiguous, because this response is made when and only when both stimuli are classified unambiguously and differently, i.e., either “AB” or “BA”.

When a “different” response is correct, it is defined as a hit. When a “different” response is incorrect, it is defined as a false alarm. Since a “different” response is defined unambiguously, a hit or false alarm is defined unambiguously also. As a result, when *c*1 and *c*2 are defined with *c*_1_ ≤ *c*_2_, the CC2a rule is completely defined.

To recap, in the CC2a rule and in each interval, a stimulus *x* can be categorized with three possibilities: “a” when *x* ≤ *c*_1_, “b” when *x* > *c*_2_, and “ambiguous” when *c*_1_ < *x* ≤ *c*_2_. Obviously this decision is independent of the stimulus sequence, which is why CC2a belongs to the case when *p*_*A**B*_ = *p*_*B**A*_. Given that the probability of both stimuli being AA but classified as “different” is: *p*_*A**A*_ = *a*_*A*_*b*_*A*_ + *b*_*A*_*a*_*A*_ = 2*a*_*A*_*b*_*A*_, and *p*_*B**B*_ = 2*a*_*B*_*b*_*B*_, from Eq. A.3, we know that $p(F) = \frac {1}{2} (p_{AA} + p_{BB}) = a_{A} b_{A} + a_{B} b_{B}$.

B.1 $$ \begin{array}{@{}rcl@{}} a_{A} b_{A}& =& {\Phi} \left (c_{1} + \frac{d'}{2} \right) \left( 1 - {\Phi} \left( c_{2} + \frac{d'}{2} \right) \right),\\ a_{B} b_{B} &=& {\Phi} \left (c_{1} - \frac{d'}{2} \right) \left( 1 - {\Phi} \left( c_{2} - \frac{d'}{2} \right) \right),\\ \therefore p(F) &= &{\Phi} \left (c_{1} + \frac{d'}{2} \right) \left( 1 - {\Phi} \left( c_{2} + \frac{d'}{2} \right)\right) \\&&+ {\Phi} \left (c_{1} - \frac{d'}{2} \right) \left( 1 - {\Phi} \left( c_{2} - \frac{d'}{2} \right)\right). \end{array} $$

B.2 $$ \begin{array}{@{}rcl@{}} &\because&  c_{1} \leq c_{2},\\ &\therefore & {\Phi} \left (c_{1} + \frac{d'}{2} \right) \leq {\Phi} \left( c_{2} + \frac{d'}{2} \right),\\ &\therefore & {\Phi} \left (c_{1} + \frac{d'}{2} \right) \left( 1 - {\Phi} \left( c_{2} + \frac{d'}{2} \right)\right) \\&&\leq {\Phi} \left (c_{1} + \frac{d'}{2} \right) \left( 1 - {\Phi} \left( c_{1} + \frac{d'}{2} \right)\right) \\ && \leq \left( \frac{1}{2} \right)^{2}.\\ &\therefore  & a_{B} b_{B} \leq \frac{1}{4}.\\ &\therefore  & p(F) = a_{A} b_{A} + a_{B} b_{B} \leq \frac{1}{2}. \end{array} $$

Given that CC2s and CC1 are both special cases of CC2a, it follows that this proof applies to CC2s (when − *c*_1_ = *c*_2_) and CC1 (when *c*_1_ = *c*_2_) also. In particular, when *c*_1_ = *c*_2_ = 0, it is the optimal independence rule, which is now proved to have a restricted *p*(*F*) ≤ 1/2.

This CC2a rule can also be extended mathematically to overcome the restriction that *p*(*F*) ≤ 1/2. Recall that CC2a was defined in Petrov (2009) as: If a stimulus *x* ≤ *c*_1_, then *x* is labeled as “A”. Otherwise if *x* > *c*_2_, *x* is labeled as “B” (*c*_1_ ≤ *c*_2_). A “different” response will be made iff one stimulus is unambiguously labeled as “A” and the other unambiguously as “B”. The proposed extension is two-fold: (1) When *c*_1_ ≤ *x* < *c*_2_, respond “different” regardless of how the other stimulus *x*_2_ is labeled (opposite to the original definition). (2) When *c*_2_ ≤ *c*_1_ such that *x* is labeled both as “A” and “B” when *c*_2_ ≤ *x* ≤ *c*_1_, then respond “same” regardless of how *x*_2_ is labeled. Under these extensions, one can see that, at one extreme, when $c_{1} \rightarrow -\infty $, and $c_{2} \rightarrow +\infty $, then the response should always be “different” such that $p(F) \rightarrow 1$ and $p(H) \rightarrow 1$. At the other extreme, when $c_{1} \rightarrow +\infty $, and $c_{2} \rightarrow -\infty $, all stimuli will be double labeled as both “A” and “B” such that $p(F) \rightarrow 0$ and $p(H) \rightarrow 0$. As a result, the ROC covers the entire range and *p*(*F*) > 1/2 will be possible.

Such an extension may be mathematically “natural,” but not necessarily so psychologically. For example, Petrov (2009) considered it psychologically natural for two uncertain stimuli to be perceived as “same”. As far as we know, such an extension has not been proposed as a psychological decision rule, possibly because of the “same” response bias when stimuli were ambiguous. For example, Bamber (1969) postulated that the “same” decision was processed in parallel, whereas the “different” decision was processed in serial. This is termed the “fast-same” effect and suggests that “same” and “different” may not be symmetric psychologically, even if they are symmetric mathematically (see also Egeth (1966)). Testing this extension is hence beyond the scope of the current study, but we will address this in the future study when participants are trained in multiple sessions.

## Appendix C: Verifying CC2a and CC1 model fitting

Recall that the independence rule is a special case of the covert classification model with one parameter. When this parameter, which is the decision criterion for each of the two stimuli, is unbiased, the model is called the independence rule. When this parameter is systematically varied in $(-\infty , +\infty )$, a linear ROC is obtained in the Z-space (or Z-ROC) whose slope is one (MacMillan and Creelman, 2005). We have empirically verified (Figs. 2 and 3) that the slopes of the human *Z*-ROCs were smaller than one for both the 8^∘^ and (after chance participants were excluded) 4^∘^ participants. We can now, based on this empirical result, independently verify whether our model fitting will give rise to the same conclusion, with the covert classification model with two parameters (CC2a) and with one parameter (CC1).

Since we have proven that, for CC2a and CC1, *p*(*F*) ≤ 1/2, we will only use the human data with *p*(*F*) ≤ 1/2 to fit the models. It also turned out that, by adding an additional Euclidean distance calculation between human and model (*p*_*A**A*_ + *p*_*B**B*_, *p*_*A**B*_ + *p*_*B**A*_) that is equivalent to insisting that (*p*(*F*),*p*(*H*)) fit well, the best and second best-fitting *d*^′^ values could better separate apart. As a result, all subsequent model fittings incorporated this additional constraint.

Since human data will be used only if *p*(*F*) ≤ 1/2, where *p*(*F*) = 1/2(*p*_*A**A*_ + *p*_*B**B*_), each of the 10 experimental sections had two to four 4-D data points that satisfied this constraint. As a result, each participant contributed on average 30 4-D data points for the model fitting. During the model fitting, an exhaustive search was conducted within the range of *d*^′^∈ [0,2.5], with a step size of Δ*d*^′^ = 0.05.

The consequence of the model fitting was that residuals were obtained as a function of the four *p*_*X**Y*_ dimensions, and of the rating criteria (two to four levels, since *p*(*F*) needed to be ≤ 1/2). There are two ways to analyze the residual data. The first is to check the magnitudes of the residuals. Obviously, the larger the residuals are, the poorer the fitting is. The second way is to check whether the residuals are evenly distributed across different *p*_*X**Y*_ dimensions and rating criteria. One can argue that even if residual magnitudes are large, but if the residuals are reasonably evenly distributed across various dimensions, then the model fitting is unbiased and has captured the mean values of human performance.

Since the participants in the current study were non-experts, we expected that the residuals could be large. Consequently, we focused our analysis on the second aspect, namely whether the residuals were evenly distributed across *p*_*X**Y*_ dimensions and rating criteria. To accomplish this, we restricted the rating data to the two smallest *p*(*F*) values so that there would be nearly no empty entries and an ANOVA was possible. Because there were only two levels of rating data used, our emphasis would be on the four *p*_*X**Y*_ dimensions to see whether residuals evenly distributed across these four dimensions. Figure 8 shows the means of residuals across the four dimensions of *p*_*X**Y*_, *X*, *Y* ∈{*A*, *B*} and the two lowest rating criteria for the two models, CC2a and CC1.

Since this is verification of the results in “Human behavioral results”, where data from 26 8^∘^ participants, and 18 4^∘^ participants (whose accuracies were > 0.52) were used, the same participants’ data were used here. We first analyzed the residuals from the CC2a model fitting to a 4 × 2 ANOVA with *p*_*X**Y*_ and rating criterion as the main factors. The main effect of *p*_*X**Y*_ was significant, *F*(3,129) = 10.64,*p* = 3 × 10^− 6^. The main effect of rating criterion did not reach significance, *F*(1,43) = 2.02,*p* = 0.16. The interaction was significant, *F*(3,129) = 5.67,*p* = 0.0011.

A similar analysis using the residuals from the CC1 model fitting gave rise to all significant effects: *p*_*X**Y*_, *F*(3,129) = 33.85,*p* = 3.33 × 10^− 16^; rating criterion, *F*(1,43) = 9.52,*p* = 0.0035; interaction, *F*(3,129) = 2.95,*p* = 0.035. Taken together, the residuals in both CC2a and CC1 model fittings were unevenly distributed across the four *p*_*X**Y*_ dimensions. The results suggest that these two models could not well explain the human data. Such conclusion is consistent with that obtained in “Human behavioral results” using completely different analysis methods.

## Appendix D: Comparing between CC2s and DF1 model fittings

In Petrov (2009), the best fitting *d*^′^ using the CC2s model was mathematically predicted to be smaller than that using the DF1 model (*d**C**C*2*s*′<*d**D**F*1′), since CC2s closely approximates the optimal model (and is therefore more efficient using the signal). Here, we verified this mathematical prediction, independently from the mathematical constraint that *p*(*F*) ≤ 1/2 for the CC2s model, which is a special case of CC2a.

Figure 9 shows, the best fitting human ROC in *p*-coordinates (since all data are within the 1 × 1 square), the best fitting CC2s ROC, human data, and the resultant CC2s *d*^′^. There were 11 panels because 11 participants’ data were potentially explainable by CC2s and DF1. A similar fitting procedure was applied using the DF1 model. Importantly, the corresponding best fitting DF1 *d*^′^ was also obtained. According to the mathematical prediction, *d**C**C*2*s*′ < *d**D**F*1′. Among the 11 participants, eight confirmed this inequality. The remaining three showed zero difference, the *d*^′^ fittings for two of the three were both *d*^′^ = 0. We conclude, separate from the *p*(*F*) ≤ 1/2 constraint, that the mathematical prediction that *d**C**C*2*s*′ < *d**D**F*1′ was consistent with the human data.

## Appendix E: Comparing RC2a and LR2 rules

We demonstrate here that the conjectured approximate equality between the RC2a and LR2 models is very limited, both mathematically and experimentally, not to say *p*(*F*) ∈ [0,1/2] for RC2a whereas *p*(*F*) ∈ [0,1] for LR2.

Petrov (2009) stated that the LR2 rule could be approximated by the RC2a rule. However, this should only be the case when the two rules give rise to similar hit and false alarm rates. As proved earlier, *p*(*F*) ≤ 1/2 for RC2a. Yet, LR2’s *p*(*F*) covers the full range of [0,1]. This full range can be seen by the following example:

$p(F) = 1 - {\Sigma }_{i=1}^{2} \left (1 - {\Phi } \left (\frac {ln \left (\beta \right )}{d'} + (-1)^{i} \frac {d'}{2} \right ) \right )^{2}$, where *β* > 1. Assuming that *d*^′^ > 0, when $\beta \rightarrow +\infty , \frac {\ln \left (\beta \right )}{d'} \pm \frac {d'}{2} {\rightarrow +}\infty $, then ${\Phi } \left (\frac { \ln \left (\beta \right )}{d'} \pm \frac {d'}{2} \right ) {\rightarrow } 1$. Therefore, $p(F) \rightarrow 1$.

Figure 10 shows direct comparison between ROC’s of RC2a and LR2 when they share the same *d*^′^.

## Appendix F: Comparing CC1 and CC2a model fittings with human data

Despite the fact that *p*(*F*) ≤ 1/2 for both the CC1 and CC2a models, we nevertheless fitted the four candidate participants’ data with the models, as shown in Fig. 11. The purpose was to verify that the best fitting *d*^′^s by the two models were similar to each other for any given participant’s data, since CC1 is a special case of CC2a (when *c*_1_ = *c*_2_). These four pairs of *d*^′^ values were indeed similar to each other, attesting to the reasonable model fittings even though *p*(*F*) ≤ 1/2 for both models — (CC1, CC2a): (2.125, 2.175), (2.150, 2.225), (0.600, 0.537), and (0.475, 0.450) ($\chi ^{2} = 1.00 < \chi ^{2}_{critical_{v}alue}=9.49$). Note also that when *c*_1_ = *c*_2_ = 0, both models become the optimal independence rule.

In addition, for these two models, Petrov (2009) stated that (*p*_*A**B*_ − 0.5)^2^ = (*p*_*A**A*_ − 0.5)(*p*_*B**B*_ − 0.5). Given that *p*_*A**B*_ = *p*_*B**A*_, the equation becomes (*p*_*A**B*_ − 0.5)(*p*_*B**A*_ − 0.5) = (*p*_*A**A*_ − 0.5)(*p*_*B**B*_ − 0.5). We also checked this equality among the four participants’ data, as follows. We checked the *χ*^2^ by assuming that either all five criteria were used or only the middle criterion was used, per experimental block. Two of the four participants data, HJL and LJJ, rejected the equality in both *χ*^2^ tests.

## Appendix G: Model comparison between CC2s, CC2a, and CC1

Via model simulations, we observed that, as a model with two parameters (*c*_1_, *c*_2_), CC2a takes up a cloud of model datum points. One of its special cases, CC2s—defined as the two criteria *c*_1_ + *c*_2_ = 0, has its ROC on the top boundary of the cloud. The other of CC2a’s special case, CC1—defined as *c*_1_ = *c*_2_, has its ROC at the bottom boundary of the same cloud. Figure 12 shows the three models’ ROCs with *d*^′^ = 1, 1.5, and 2.

## Appendix H: Model comparison between RC2s and RC2a

Similar to the last section, we also compared model performance between RC2s and RC2a, where RC2s is a special case of RC2a, which has two symmetric criteria about the midpoint between the two distributions. Figure 13 shows these models’ performance with *d*^′^ = 1, 1.5, and 2. Note that in each panel, the RC2s ROC is on top of the RC2a data clouds (RC2s = CC2s).

## Footnotes

1 In the rest of the analysis, these ten participants would be still included. This is because the accuracy, as a single number, cannot sufficiently constrain the distribution of *p*_*X**Y*_ (where *X*, *Y* ∈{*A*, *B*}) across rating criteria for model-fitting purpose, even though chance performance tends to favor *p*_*A**B*_ + *p*_*B**A*_ = *p*_*A**A*_ + *p*_*B**B*_ on average.

2 It should be noted that the model comparison here is under the assumption that a human ROC from a rating experiment can be similarly obtained from a number of binary same-different tasks each with a different criterion or criteria. We understand that there is disagreement against this assumption (Van Zandt, 2000; Balakrishnan, 1999), but believe that the best way to address this controversy is empirical. Accordingly, we ran five additional participants in a binary same-different experiment that was otherwise identical, except the participants were instructed to reply “same” if and only if they were absolutely certain. There was no feedback either (since feedback would nudge the criterion to the middle). The new [*p*(*H*),*p*(*F*)] are: [0.78, 0.67], [0.68, 0.58], [0.84, 0.70], [0.72, 0.62], and [0.85, 0.65]; clearly indicating that human criterion or criteria could give rise to *p*(*F*) > 1/2.

3 Note that a hit here is defined as *p*(“different”|*A**B* ∪ *B**A*), which was defined as correct rejection in Irwin and Hautus (1997).

4 The order of the cases in Table 2 is different from that in Petrov (2009) because presenting simpler, one-parameter models first may aid understanding of the subsequent two-parameter models.

5 Petrov (2009) conjectured that RC2a = LR2, but that LR2 was an unlikely model for human performance
